# Successful Implementation of a Perioperative Glycemic Control Protocol in Cardiac Surgery: Barrier Analysis and Intervention Using Lean Six Sigma

**DOI:** 10.1155/2011/565069

**Published:** 2011-09-06

**Authors:** Elizabeth A. Martinez, Raul Chavez-Valdez, Natalie F. Holt, Kelly L. Grogan, Katherine W. Khalifeh, Tammy Slater, Laura E. Winner, Jennifer Moyer, Christoph U. Lehmann

**Affiliations:** ^1^Department of Anesthesia, Critical Care and Pain Medicine, Massachusetts General Hospital, Harvard Medical School, 55 Fruit Street, GRB 4-44, Boston, MA 02114, USA; ^2^Division of Neonatology, Department of Pediatrics, Texas Tech University Health Sciences Center, 701 West 5th Street, Odessa, TX 79765, USA; ^3^Division of Neonatology, Department of Pediatrics, Johns Hopkins Medicine, 600 North Wolfe Street, Nelson 2-133, Baltimore, MD 21287-3200, USA; ^4^Department of Anesthesiology, West Haven Veterans Affairs Medical Center, Yale University School of Medicine, 333 Cedar Street, TMP 3, P.O. Box 208051, New Haven, CT 06520-8051, USA; ^5^Department of Anesthesia, Medical University of South Carolina (MUSC), 25 Courtenay Drive, Suite 4200, MSC 240, Charleston, SC 29425-2400, USA; ^6^Department of Surgery, Johns Hopkins Medicine, 600 North Wolfe Street, Blalock 655, Baltimore, MD 21287, USA; ^7^Cardiac Surgical ICU, Department of Nursing, Johns Hopkins Medicine, 600 N. Wolfe Street, Halsted 600, Baltimore, MD 21287, USA; ^8^Director Lean Sigma Program, Center for Innovation in Quality Patient Care, Johns Hopkins Medicine, 601 North Caroline Street, Suite 2080, Baltimore, MD 21287-0765, USA

## Abstract

Although the evidence strongly supports perioperative glycemic control among cardiac surgical patients, there is scant literature to describe the practical application of such a protocol in the complex ICU environment. This paper describes the use of the Lean Six Sigma methodology to implement a perioperative insulin protocol in a cardiac surgical intensive care unit (CSICU) in a large academic hospital. A preintervention chart audit revealed that fewer than 10% of patients were admitted to the CSICU with glucose <200 mg/dL, prompting the initiation of the quality improvement project. Following protocol implementation, more than 90% of patients were admitted with a glucose <200 mg/dL. Key elements to success include barrier analysis and intervention, provider education, and broadening the project scope to address the intraoperative period.

## 1. Introduction

Methods to develop evidence-supported practices for quality improvement projects became prevalent in the 1990s, yet, two decades later, significant gaps persist in translating the best evidence into practice [1]. This is particularly true for complex disease states (such as insulin resistance) coupled with complex procedures (such as cardiac surgery). In addition, the majority of clinical research has focused on understanding disease processes and identifying effective therapies, yet there is relatively little emphasis on the implementation side. The available literature tends to recommend using system-wide changes to alter provider behaviors [2]. System change is becoming increasingly important as public reporting of compliance with evidence-based protocols and “pay-for-performance” reimbursement gain ground. Yet, ultimately, to change provider behavior, we must first understand facilitators and barriers. To this end, Cabana and colleagues [3, 4] explored physician barriers to the implementation of best practices and proposed general approaches to modifying individual provider behavior. Unfortunately, such generalizations do not address the unique complexities of the intensive care unit (ICU), where a confluence of provider preferences, patient comorbidities, and system factors make an interdisciplinary local approach more effective. 

Different methods have been proposed to facilitate clinical guideline implementation, for example, Plan-Do-Study-Act, total quality management, and continuous quality improvement [5, 6]. One performance improvement approach that has proven effective in driving high reliability is Lean Six Sigma (LSS) [7–9]. The LSS method incorporates data-driven methods which focus on reducing defects and undesirable variation within a process. 

Because LSS is designed to function in complex environments, the methodology is promising for application in healthcare systems. The LSS approach addresses operational aspects including change management, identification of barriers, and other limiting factors; it also provides straightforward analytical tools and improvement monitoring methods [10, 11]. Because the evidence strongly supports achieving glycemic control in cardiac surgical patients to improve outcomes [12, 13], we selected the LSS as a strategy for implementing an insulin protocol in a cardiac surgical ICU (CSICU) in a large academic hospital. We hypothesized that the comprehensive LSS approach would generate a substantial and sustainable improvement in perioperative glucose control, specifically that we would increase the number of CSICU admissions with a glucose <200 mg/dL, decrease the time to control and percentage of patients in control at 6 hours post-ICU admission (defined as 80–110 mg/dL), and improve the overall time in control while not increasing hypoglycemic events. While published reviews have proposed “pointers” for implementation of an ICU insulin protocol [14], this paper is the first to detail how LSS methods can be used to improve glycemic control in a complex patient-care setting. 

**Figure 1:**
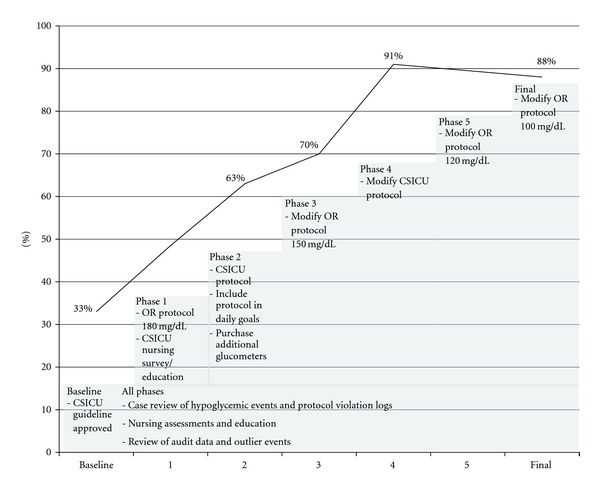
Prospective audit data by implementation phase of CSICU Lean Six Sigma glucose control project.

A glucose control guideline was developed in 2002 for the CSICU. In June 2003, 6 months after rollout, a multidisciplinary team (nursing, surgery, anesthesiology, and hospital quality improvement) conducted a chart audit (Figure 1) and concluded that glycemic control remained poor despite the compelling evidence in the literature and the CSICU's new guideline. Consequently, a formal LSS-structured improvement initiative was launched. This paper presents the process of identifying barriers and improving clinician compliance with a new formal protocol, as well as our practical experience using LSS to identify and overcome these barriers. While suggested target glucose levels have changed since the protocol was originally designed [15], the methodology and performance monitoring presented here remain relevant. 

## 2. Methods

In 2003, as part of a performance improvement project, we evaluated the current process of glycemic control in the CSICU of a tertiary care center. To manage the project, data were collected prospectively with periodic chart reviews using standardized tools during the study period June 2003–2007. After Institutional Review Board (IRB) approval, additional electronic data were captured retrospectively for this publication according to the processes outlined next. 

### 2.1. Lean Six Sigma Methods

The multidisciplinary team sought to improve glycemic control using the LSS DMAIC framework (define, measure, analyze, improve, and control) in the tertiary care center's CSICU. Each step of the DMAIC process is detailed herein.

**Define**

The team developed a project charter to capture the magnitude of the problem, and define the local contributing factors and opportunities for improvement. A baseline chart audit was conducted (KK) which suggested that the majority of patients were severely hyperglycemic upon CSICU admission (33% of patients with admission glucose <200 mg/dL, Figure 1). Thus, for the initial project charter, we defined five measurable goals (outcome measures, or “Y”s) of the improvement program as follows.

1. Improve glucose control upon admission to the CSICU: increase the number of patients with glucose <200 mg/dL on admission to the ICU from 33% to 73%; (a 40% increase). (This target was selected according to LSS principles: the goal was to improve performance by 80%; thus, the LSS recommendation for the initial intervention is to set a preliminary goal halfway to the ultimate objective.)
2. Improve glucose control following admission to the CSICU: increase the proportion of patients in control (glucose between 80 and 110 mg/dL) at 6 hours post-CSICU admission from 0% to 20%; (a 20% increase).
3. Reduce the time to control (measured in hours from admission).
4. Improve the mean time-weighted glucose level (TWGL).
5. Reach and hold a steady state of hypoglycemic events <2% as defined by the existing hospital tracking system (percentage of total glucose values).

**Measure**

The performance improvement interventions and thus the measurement phases were iterative (Figure 1, Table 1). This work was completed during monthly QI team meetings and in small working groups.

**Figure 2:**
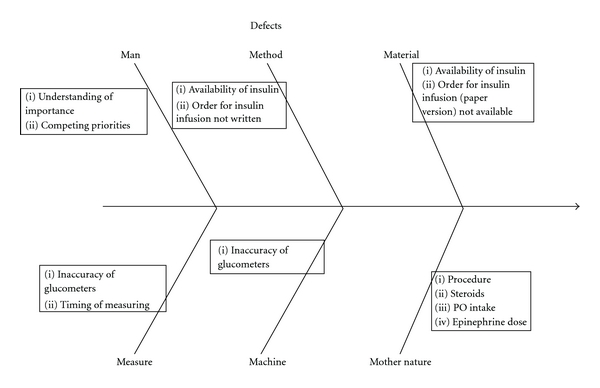
Fishbone diagram of glucose protocol implementation process.

**Figure 3:**
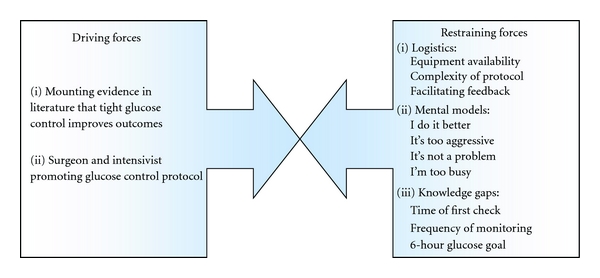
Force field analysis based on CSICU nursing assessment.

- *Identification of Baseline Capability (or “Y”s) and Following Protocol Modifications (Phases of Intervention)*. Glucose values and associated time points post-ICU admission, deviation from protocol, and presence of vasoactive agents were collected via periodic real-time chart audits using a standardized instrument. Data were then entered into an Excel spreadsheet. Since both point-of-care glucometer data and core laboratory data were used by bedside practitioners to make insulin adjustments, both sources of glucose measurement were included in the analysis.
- *Identification of Key Contributing Factors (or “X”s) to Inadequate Control*. Possible factors shown (or perceived) to be contributing to poor control were generated during QI meetings and augmented by a nursing survey (see next, assessment of current practice). These factors are depicted in a fishbone (cause-and-effect) diagram (Figure 2) and force field analysis (Figure 3).
- *Assessment of Current Practice*. In addition to the baseline chart audit, a QI team member (NH) established a focus group with the ICU nursing staff and developed an evaluation tool (survey) to identify the CSICU nurses' state of knowledge regarding the evidence for glucose control, understanding of the existing guideline, and barriers to protocol implementation. This evaluation tool identified that staff believed the glucose control guideline was “just a research project” that one of the physicians developed rather than an evidence-based recommendation to improve patient outcomes. Furthermore, nurses believed that the absence of an intraoperative glucose management strategy confirmed the lack of importance of the issue. In addition, the existing CSICU guideline lacked clarity on many aspects (Figure 3). Fewer than half of the nurses (41%) felt the current protocol was successful; many nurses felt adherence to the CSICU insulin guideline impeded rather than improved their chance of achieving glycemic control. These barriers are represented in Figure 3 and categorized as logistical, mental models and knowledge gap restraining forces. An additional criticism identified through the focus groups was that none of the bedside nurses participated in the development of the existing guideline and no usability testing had been performed.
- *Development of a Process Map for Implementing an Insulin Protocol*. A process map of the steps required by nurses to implement the current glucose guideline was developed; another process map identified time-limiting steps and potential barriers to successful implementation of the formal protocol. The nursing staff identified key issues including the following: (1) an order allowing them to follow the glucose control guideline was frequently not written at admission (partly owing to ambiguity of which patients qualified for the protocol); (2) delays or shortages of insulin infusion pumps from pharmacy allowed glucose levels to climb following admission to the ICU.

**Analyze**

The key contributing factors to poor glucose control, the findings from the front-line provider survey and interviews, barriers, and process maps were discussed at the QI team meetings. Data from prospective audits of glucose control were reviewed on an ongoing basis to inform changes in the insulin protocol (Figure 1). Data for the audits were obtained from the electronic records. Data abstracted included glucose values, concomitant vasoactive infusions, insulin dosing, and compliance with the protocol including time of lab draws.

### 2.2. Statistical Analysis

**Figure 4:**
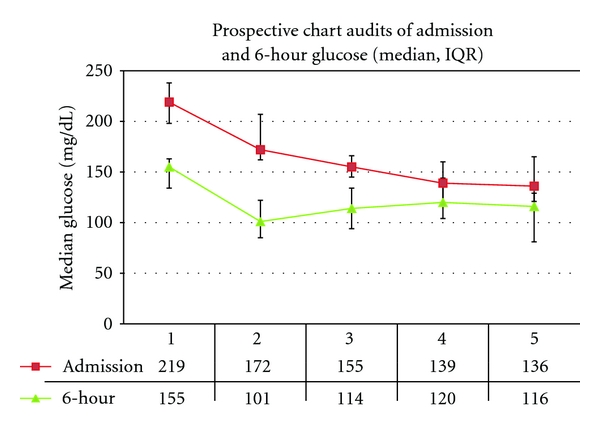
Informing protocol modification with real-time audit data: initial and 6-hour glucose. Audits 1–5 reflect targeted data collection at Baseline (June 2003); Phase 2 (June 2004); Phase 3 (January 2005); Phase 4 (June 2005); Final (December 2007).

Qualitative analyses were performed on findings from focus groups. Real-time chart audit data were reviewed informally with individual nurses and practitioners to keep the process moving forward. This was done on an ongoing basis. Nursing champions would discuss these data at regularly scheduled staff meetings. More formal interim reviews and real-time audits included trend analyses and case reviews to inform modifications of the glucose control protocols (Figure 4). 

For the cumulative retrospective analysis, we evaluated the following variables: time-weighted glucose level (TWGL) [16]; mean and median glucose levels for the length of stay; admission glucose level; time to glucose control (time to control) from CSICU admission (hours); percent of hypoglycemic patients and incidents per phase of intervention. (The TWGL was selected to represent the overall glycemic control of the patient because it takes into account the increased clinical likelihood for patients with an abnormal value to have more frequent rechecks, while those with normal values have fewer repeat measures. Using averages or means would therefore skew data to the abnormal values.) The goal glucose on admission was <200 mg/dL and “in control” was defined as 80–110 mg/dL for the quality improvement time period. Data for the retrospective analysis were obtained through abstraction of lab values from the electronic record. For continuous variables, data distribution and variance homogeneity were determined by the Shapiro-Wilk and Levene tests, respectively. All continuous variables included in this analysis were found to be nonnormally distributed; thus, all multiple group (phase) comparisons were made by Kruskal-Wallis ANOVA and post hoc tests assuming unequal variance by Tamhane's T2 test. Data are displayed with box-and-whisker plots in which the box and line inside the box represent the interquartile range (IQR, 25th and 75th percentile) and the median, respectively. Whiskers extend up to the last data point available within 1.5 times the IQR from the median. Outliers were removed from the plots in order to allow marks to be placed. For binomial variables, data were analyzed by Chi square test in all cases and represented as bar graphs where appropriate. Statistical significance was defined as *P* < 0.05. Analyses were performed using IBM SPSS Statistics 18 software (IBM Corporation, Somers, NY, USA).

**Improve**

Based on findings from interim reviews during the measure and analyze phases, a multipronged and sequential approach was taken to improve glucose control. The OR and ICU protocols were modified periodically; project phases and key interventions are noted in Figure 1 and Table 1.

**Figure 5:**
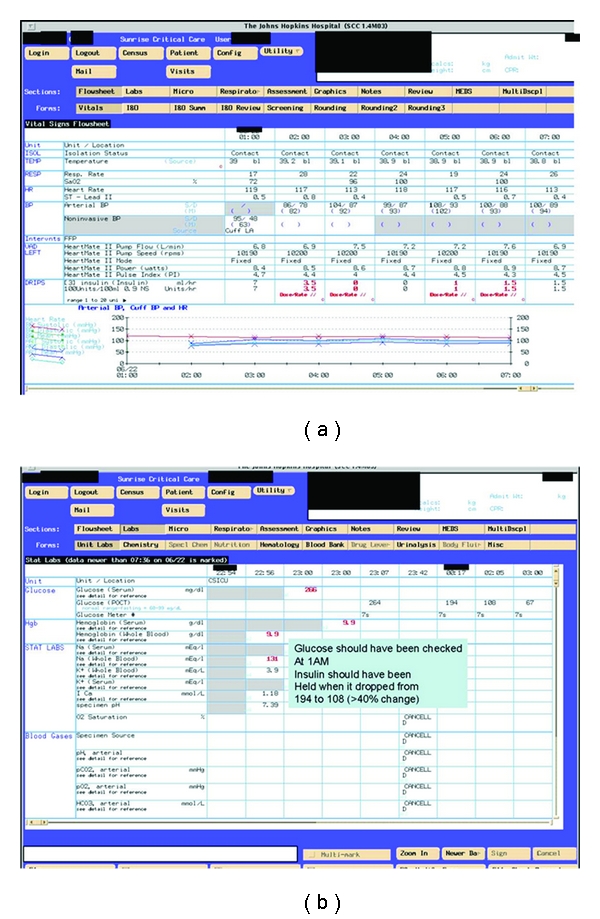
Sample case for review during “CICU Glucose Rounds.”

- *Implemented formal glucose control protocol in the OR during Phase 1*, thus driving the intervention focus further “upstream” from the CSICU. Prior to implementation, the CSICU data and proposed anesthesia-developed OR protocols were reviewed with the entire cardiac anesthesia division. Given the concerns about the lability of the intraoperative period and the heightened risk of unrecognized hypoglycemia in patients under anesthesia, the group agreed to an initial target CSICU admission glucose of <200 mg/dL. In order to increase the comfort level of the providers, the protocol was initially developed such that insulin would be initiated in the OR for glucoses >180 mg/dL with the intention of frequent assessment for safety.
- *CSICU nursing education was formalized in Phase 2*. Multiple educational events included distribution of the new formal protocol and integrating the rationale behind the glycemic control protocol during nursing skills days and new staff orientations.
- *Implemented formal glucose control protocol in the CSICU with ongoing assessment plan and feedback to nursing staff in Phase 2*. A complete redesign of the glucose control protocol was undertaken with CSICU nursing input. Introduction of the new formal protocol was planned to coincide with a previously planned nursing skills day to generate enthusiasm and allow staff to ask questions. Following implementation, five intermittent real-time audits were performed (Figures 1 and 4) to assess compliance with the new formal protocol and to identify potential risks including hypoglycemic events. This process included real-time discussions with bedside providers to gain continued insight into the limitations of the protocol and its successful implementation, updates at bimonthly staff meetings, and posting of data in central areas. The issue of glucose control was added to the CSICU daily goal sheet [17] to make certain that a discussion about glycemic control was included during morning rounds. “Glucose rounds” were also implemented during which time the CSICU nurse manager or charge nurse would lead a discussion among the nursing staff regarding a particularly challenging case or would review data pertaining to achieving glycemic control (sample case review provided in Figure 5). Early on, the focus of the audits and glucose rounds was to emphasize the importance of following the protocol so that it could be accurately evaluated. Frequently nursing staff would state that the protocol was too aggressive and resulted in hypoglycemic episodes. However, through a review of specific cases, it was identified that many of these events occurred because the protocol had not been followed accurately—most frequently due to failures in glucose monitoring which the nurses ascribed to lack of time or availability of glucometers (Figure 3). This finding resulted in purchasing additional bedside glucometers so that nurses would not have to leave a patient's room in order to carry out the test.

**Control**

In order to maintain the momentum, education on glucose management was incorporated into the orientation of all new nursing staff. In addition, ongoing education and data updates were included in bimonthly staff meetings. In the surgical suite, the insulin protocol was posted in all ORs for easy access and included in resident orientation materials. In addition, following the final audit, admission glucoses were tracked on a monthly basis (data not shown) as an extension of the performance improvement project to quickly identify any deterioration in performance. Each patient who did not meet set criteria was investigated.

## 3. Results

**Figure 6:**
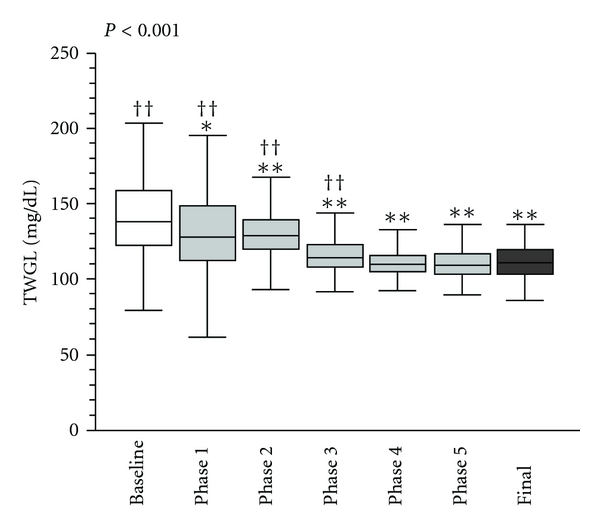
TWGL by phase of intervention (retrospective data analysis). Box-and-whisker plot represents TGWL (mg/dL) by phase. *P < 0.05 and **P < 0.01 (versus baseline) and ^†^ *P* < 0.05 and ^††^ *P* < 0.01 (versus final phase).

**Figure 7:**
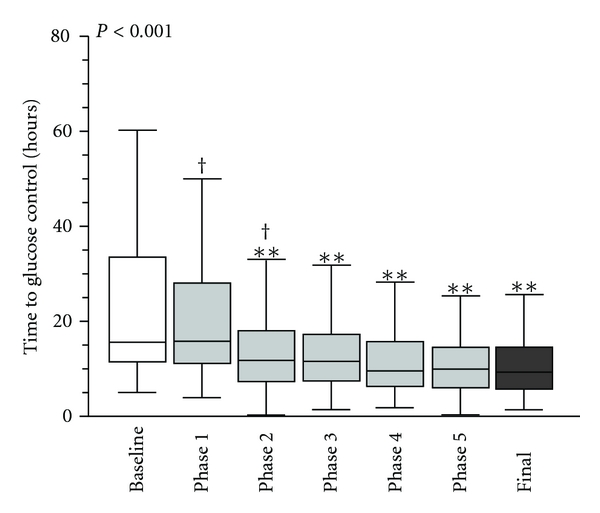
Time to control (retrospective data analysis). Box-and-whisker plot represents time to control (mg/dL) by phase. *P < 0.05 and **P < 0.01 (versus baseline) and ^†^ *P* < 0.05 and ^††^ *P* < 0.01 (versus final phase).

The implementation timeline and five real-time audit data are presented in Figures 1 and 4. Data from the real-time audits informed the ongoing quality improvement process while the retrospective analysis was used to evaluate the overall impact of the LSS project. The seven data collection periods (Baseline, Phases 1–5, and Final) are included in the retrospective analysis (Table 2 and Figures 1, 6, and 7).

### 3.1. Real-Time Chart Audits

Five prospective audits were performed (Figures 1 and 4). At audit no. 1, only 33% of patients had an admission glucose of <200 mg/dl, compared to 88% in audit no. 5. At audit no. 1 (baseline), no patients had a glucose <110 mg/dL at 6 hours compared to 41% in audit no. 5. Additional results of the prospective audits are shown in Figure 4 (admission and 6-hour glucoses). An example of the instruments used for “CSICU glucose rounds” is shown in Figure 5.

### 3.2. Retrospective Analysis

We performed a global retrospective analysis of 1892 patients who were admitted to the CSICU during the phases described in Table 1. At the completion of the LSS program, glucose data were abstracted from the electronic medical record. There were a total of 81,333 glucose checks and a mean (±SD) of 8(±5) glucoses per patient-day. The average number of glucose measurements/patient/day increased significantly over the study period from 3(±4) at baseline and phase 1 to 12(±4) during the final phase (*P* < 0.001) (Table 2). Using the retrospective data, at baseline, 76% of patients had an admission glucose of <200 mg/dL compared to 94% in the final phase (*P* < 0.001), an improvement of 18%. No patients had a glucose <110 mg/dL at 6 hours at baseline compared to 11% in the final phase (*P* < 0.001). The impact of the protocol is substantiated by two additional outcomes: (1) TWGL from a median of 138 (32) to 109 (13) (Figure 6) and, (2) significant improvement in the time to achieving glucose control from a median (IQR) of 16 (22) hours at baseline to 9 (4) hours in the final phase (Figure 7). 

The impact of the sequential changes in the OR protocol is evident by the data throughout Table 2. While the number of hypoglycemic events remained low (1.7% at baseline and 0.9% at Phases 5 and Final), the proportion of patients with a hypoglycemic event increased significantly from 9.5% at baseline to 19% at final (*P* < 0.001).

## 4. Discussion

This work represents the use of LSS methodology to successfully implement a glycemic control protocol in a CSICU. While many articles have highlighted the evidence to support perioperative glucose control in cardiac surgical patients [15, 18–21] and some have included actual protocols utilized [18, 22, 23], we are unaware of any prior articles detailing the multifaceted process of implementing a comprehensive insulin protocol in a critical care setting. This paper demonstrates that LSS methodology can be an effective tool in achieving new process implementation. In particular, we emphasize how barriers to success can be successfully identified and overcome.

Implementation of an insulin protocol in an ICU setting is challenging due to the complex nature of both the guideline and the environment. Achieving success with glucose control is difficult for several reasons. First, providers are skeptical about the benefits of maintaining glycemic control and express concerns about increasing the risk for hypoglycemia, with its potentially devastating sequelae. Second, glucose management is a complex, multistep process with independent probabilities of failure associated with each step and patient coupled with a dynamic set of variables persistently changing a patient's insulin requirements. Third, achieving optimal glucose control is a labor-intensive process, requiring human and equipment resources that are often scarce in a high-acuity environment. While targeted glucose values have been modified over the years [15, 24], the approach to addressing barriers and optimization of glucose management are unchanged. By using LSS tools, we developed unforeseen insights into the problem of achieving glycemic control in the CSICU, such as the importance of beginning the process of glucose management in the OR and incorporating the knowledge and concerns of key stakeholders (frontline providers) into the design and implementation of a glucose protocol. 

Several factors must be considered when interpreting our data. First, we chose to use LSS methodology to achieve our glycemic control goal and were successful with this method. However, we did not compare this methodology to other established QI methodologies such as PDSA and total quality management [5, 6], and it is likely that these other approaches could be similarly effective. The advantage offered by LSS was that many of the QI team were trained in this method and thus shared the same framework for discussing the problem. We felt this was an important element, and when designing quality improvement initiatives it is essential for all members of the team to share the same mental model for the approach. Furthermore, we felt the LSS tools would be useful in identifying local barriers to success, which we believed *a priori *were significant contributing factors to the failure to achieve glycemic control with the initial implementation of a glucose control guideline. Such specific tools are lacking in other general quality improvement methodologies. Second, our report highlights the variation in outcomes (glucose control) when performing audits in real-time as compared to collecting data on a larger ICU patient population retrospectively. For example, our real-time audits underestimated the proportion of patients with glucose levels <200 mg/dL (33% compared to 76% in the retrospective cohort). While the real-time data is invaluable, this is a potentially important aspect to consider when implementing new protocols—the baseline audit is a snapshot in time. Institutions may wish to couple a retrospective review for a similar time period (if seasonality is likely to be a factor) with a real-time assessment to get a balanced picture of current performance. The discrepancy we found is likely due to sampling error as the audit was conducted based on a census prevalence (20 patients in the CSICU) compared to 390 patients included in the retrospective baseline cohort; it could also reflect the random chance that higher risk patients were admitted to the CSICU the week of the audit. Incorporating data requirements into electronic health records could greatly facilitate the process of data management and analysis for QI programs. Third, recommendations for the best measure of effectiveness and risk of an insulin protocol vary widely in the literature [13, 16, 25, 26]. In our analysis, we used admission glucose, the time to control, and the TWGL and we report percent of glucoses to represent hypoglycemic events. When determining our local acceptable rate of hypoglycemia, we used the global measure of percentage of glucose checks since these data were readily available. However, this measure may be misleading since we had a notable percentage of patients with glucose values less than 60 mg/dL (with an early increase to >20% and 19% in the final phase). While we stated in our charter that we did not want to increase our rate of hypoglycemia, our baseline rate was extremely low since so few patients were on an insulin infusion prior to the implementation of this process improvement initiative. Additionally, patients on average had only 3(±2) glucose measures/day, compared to 12(±4) in the final phase of our initiative. A fourth limitation is that we elected not to collect demographic data on our CSICU patient population because our intention was to develop a protocol that was effective for all patients admitted to the CSICU, and therefore would be inherently insensitive to demographic indicators. Demographic data might have offered important insights into the predictors of hypoglycemia and poor control. The perception of the LSS team was that the complexity of the patients increased during the study period, with an increased cohort of patients with heart failure requiring higher doses of epinephrine, our first line inotrope. If we had been able to identify them, it might have yielded beneficial information for others developing protocols. Furthermore, we may have exhibited even greater relative improvements had we been able to risk adjust for patient severity and include a measure of vasoactive support. A fifth limitation is that we did not collect outcomes such as infection and mortality rates. However, the intermediate outcome of glucose control has been shown to be associated with a reduction in morbidity and mortality [15] and was more directly relevant to our stated project goal.

## 5. Conclusion

Implementation of evidence-based practices is very complex and failure prone. Evidence is necessary but not sufficient to complete implementation: the evidence provides the “what to do” but not the “how to do.” The lack of translational science providing the path from evidence to the bedside may be the reason why the literature suggests it takes one to two decades for evidence-based guidelines to be incorporated into clinical practice [1]. 

In order to deliver evidence-based practices consistently, we recommend locally developed or modified (i.e., ICU- and OR-specific) protocols, routine evaluation (and reevaluation) of barriers, and engagement of a multidisciplinary team to design intervention plans and evaluation measures. This study demonstrates the practicality and effectiveness of using LSS techniques to substantially effect change in a complex clinical environment.

## Figures and Tables

**Table 1:** CSICU glucose protocol timeline.

Phase and Date	Primary intervention	Study period
Baseline	Nursing assessment via survey. Measure admission glucose level in CSICU	1/8/03–6/27/03

Phase 1 (7/25/03)	OR protocol (begin insulin for glucose >180 mg/dL)*	8/15/03–11/29/03

Phase 2 (12/15/03)	CSICU nursing education and implementation of new CSICU glycemic control protocol	1/12/04–4/15/04

Phase 3 (5/1/05)	OR protocol (begin insulin for glucose >150 mg/dL)*	5/15/05–8/12/05

Phase 4 (9/1/05)	Introduction of revised CSICU protocol	10/7/05–12/31/05

Phase 5 (2/1/06)	OR protocol (begin insulin for glucose >120 mg/dL)*	2/16/06–6/15/06

Final (11/1/06)	OR protocol (begin insulin for glucose >100 mg/dL)*	2/19/07–3/31/07

*Goal admission glucose <200 mg/dL.

**Table 2:** Retrospective evaluation of glucose data by project phase.

		Baseline	Phase 1	Phase 2	Phase 3	Phase 4	Phase 5	Final	*P* value
Patients	*n*	390	221	267	221	204	279	310	—
Glucose datapoints	*n*	3778	2505	11063	13255	13509	18180	19043	—
Datapoints/ patient/day	Mean (±SD)	3 (±2)	3 (±2)	8 (±3)	12 (±5)	11 (±5)	11 (±5)	12 (±4)	<0.001^a^
Admit glucose (mg/dL)	Median (IQR)	160 (67)	144 (56)	161 (57)	144 (58)	136 (57)	133 (49)	124 (48)	<0.001^b^
Admit glucose <200 mg/dL	% (*n*)	76 (297)	89 (197)	81 (217)	87 (192)	90 (184)	95 (266)	94 (291)	<0.001^c^
TWGL (mg/dL)	Mean (±SD)	141 (±26)	133 (±27)	129 (±22)	116 (±12)	112 (±11)	111 (±11)	111 (±11)	<0.001^a^
	Median (IQR)	138 (32)	129 (37)	129 (20)	115 (15)	110 (11)	109 (14)	109 (13)	<0.001^b^
Time to glucose control (hr)	Median (IQR)	16 (22)	15 (17)	12 (11)	11 (10)	9 (9)	10 (8)	9 (8)	<0.001^b^
Glucose 6 h control	% (*n*)	0 (0)	0.5 (1)	7.1 (19)	4.5 (10)	5.9 (12)	8.6 (24)	11 (34)	<0.001^c^
Hypoglycemia events	% (*n*)	1.7 (63)	1.5 (38)	1.5 (168)	1.2 (161)	0.7 (90)	0.9 (160)	0.9(173)	<0.001^c^
Hypoglycemic patients	%(*n*)	9.5 (37)	10.9 (24)	21.3 (57)	23.1 (51)	21.6 (44)	16.8 (47)	19 (59)	<0.001^c^

^a^One-way ANOVA; ^b^Kruskal-Wallis ANOVA; ^c^Chi-square.

IQR: Interquartile range; Max: maximum; *n*: number of patients; SD: standard deviation; TWGL: time-weighted glucose levels.
